# Quantile Regression in Epidemiology: Capturing Heterogeneity Beyond the Mean

**DOI:** 10.3390/mps9010002

**Published:** 2025-12-21

**Authors:** Charalambos Gnardellis

**Affiliations:** Department of Fisheries and Aquaculture, School of Agricultural Sciences, University of Patras, 30200 Messolonghi, Greece; hgnardellis@upatras.gr

**Keywords:** quantile regression, ordinary linear regression, epidemiological methods, body mass index, heteroscedasticity, median regression, population health, statistical modeling

## Abstract

Ordinary linear regression is the most common approach for modeling relationships between continuous outcomes and explanatory variables in epidemiological research. However, this method relies on restrictive assumptions—normality, homoscedasticity, and linearity—that are often violated in real-world biomedical data. When these assumptions fail, mean-based estimates may obscure important heterogeneity across the outcome distribution. This study aims to illustrate the methodological and interpretive advantages of quantile regression over ordinary regression in the analysis of epidemiological data. Secondary data were derived from a cross-sectional study of 1415 healthy Greek adults aged 25–82 years. Body mass index (BMI) served as the outcome variable, while sex, age, physical activity, dieting status, and daily energy intake were considered predictors. Both ordinary and quantile regression models were applied to estimate associations between BMI and its determinants across the 25th, 50th, 75th, and 90th quantiles. Ordinary regression identified positive associations of BMI with age and energy intake and a negative association with physical activity. Quantile regression revealed that these relationships were not constant across the BMI distribution. The inverse association with physical activity intensified at higher quantiles, and the gender effect reversed direction at the upper tail, suggesting heterogeneity was not captured by mean-based models. Quantile regression provides a distribution-sensitive alternative to ordinary regression, offering insight into covariate effects across different points of the outcome distribution and serving as both a robust analytical tool and an educational framework for applied epidemiological research.

## 1. Introduction

In epidemiological research, relationships between a continuous outcome and a set of predictors are often examined using the ordinary linear regression model. This model assumes that the association between a dependent variable Y  and a set of independent variables $X_{1},X_{2},\ldots ,X_{k}\;$ is linear, and that the conditional distribution of Y  for any given values of the predictors is normally distributed with constant variance. Formally, this relationship is expressed as:

(1) $$Y_{i}=\beta_{0}+\beta_{1}X_{1i}+\beta_{2}X_{2i}+\ldots +\beta_{k}X_{ki}+\varepsilon_{i},\;i=1,\ldots ,n,$$

where $\varepsilon_{i\;}$ represents the random error term, assumed to follow a normal distribution with mean zero and constant variance, $\varepsilon_{i}∼N(0,\sigma^{2})$. Under these assumptions, the fitted regression line offers an efficient linear summary of how the mean value of Y  changes with the predictors. However, these assumptions are often violated in real-world epidemiological data, where outcomes are rarely symmetrically distributed [1,2].

### 1.1. Violations of Classical Assumptions

**Figure 1:**
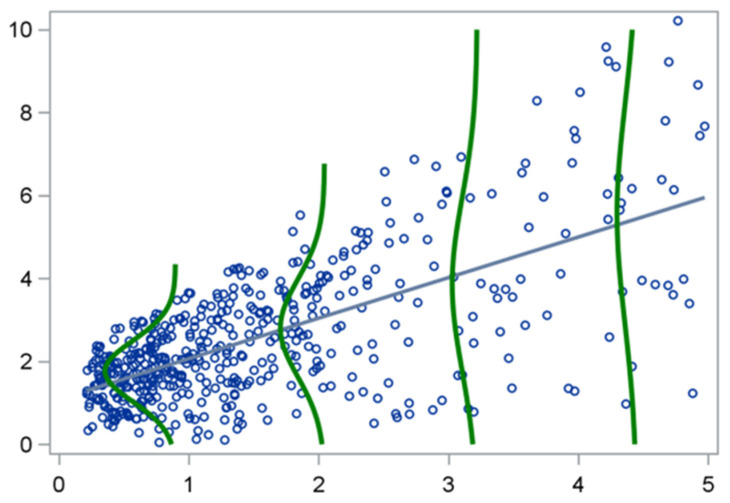
A case of simple linear regression where the assumption of homoscedasticity is violated.

Consider, for example, the data illustrated in Figure 1, which depict a simple linear regression model where the assumption of homoscedasticity (constant variance) is violated.

The regression line approximates the values of Y  fairly well when X  is small, but as X  increases, the spread of Y  becomes larger. Although each subgroup of Y  values (for a given X) may still be approximately normal, their variances differ substantially. This pattern of increasing variability—common in epidemiological and biomedical data—renders the model estimates inefficient and the inference unreliable [3]. In such situations, the mean is no longer an appropriate measure of central tendency. For example, when modeling biomarkers, hospitalization duration, or healthcare costs, data are often right- skewed, meaning that a small number of high values distort the mean. A regression model focusing exclusively on the conditional mean of Y can thus misrepresent the true nature of the relationship between predictors and outcomes [4,5,6].

### 1.2. From Mean to Quantiles

**Figure 2:**
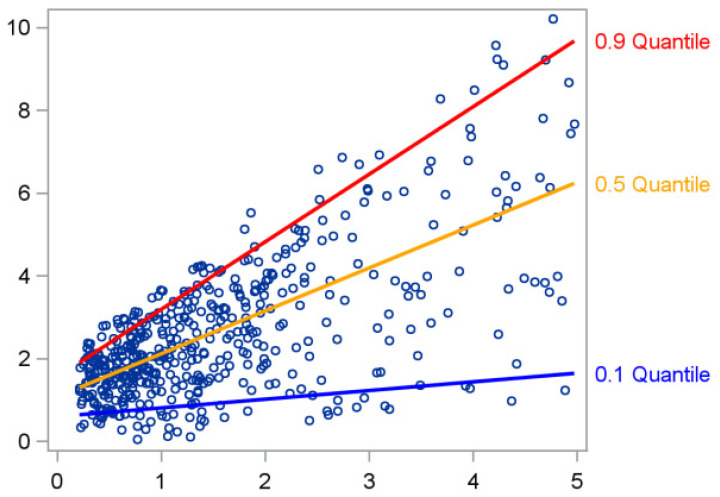
Regression lines estimating different quantiles of Y.

A more informative and robust approach involves moving beyond the mean and exploring different quantiles of the outcome distribution. Instead of fitting a single regression line that predicts the mean of Y, we can fit several regression lines that estimate specific percentiles (or quantiles)—for example, the 10th, 50th (median), or 90th percentile [7,8,9]. This idea is illustrated in Figure 2, where each line corresponds to a different quantile of Y.

While the least-squares line provides an overall average trend, the quantile regression lines reveal how the relationship between *Y* and *X* varies across the outcome distribution—steeper for the higher quantiles and flatter for the lower ones, for instance [10]. Quantiles are defined by a coefficient *τ* that takes values in the interval (0,1), indicating the proportion of the distribution below that quantile. For example, *τ* = 0.10 corresponds to the 10th percentile, while *τ* = 0.50 corresponds to the median. In the remainder of the text, the terms quantile and percentile will be used interchangeably.

### 1.3. Quantile Regression Framework

Quantile regression estimates the conditional quantile function $Q_{\tau }\left(Y_{i}|X_{i}\right)\;$ of the dependent variable as a linear function of the predictors:

(2) $$Q_{\tau }\left(Y_{i}|X_{i}\right)=\beta_{0}\left(\tau \right)+\beta_{1}\left(\tau \right)X_{1i}+\beta_{2}\left(\tau \right)X_{2i}+\ldots +\beta_{k}\left(\tau \right)X_{ki},$$

where $\tau \in \left(0,1\right)\;$ denotes the quantile of interest (e.g., τ=0.25  for the 25th percentile, τ=0.50  for the median, etc.). The parameters $\beta_{j}(\tau )$ are estimated by minimizing the asymmetric absolute loss function:

(3) $$\underset{\beta (\tau )}{min}\sum_{i=1}^{n}\rho_{\tau }(Y_{i}-X_{i}^{\prime }\beta (\tau )),$$

where

(4) $$\rho_{\tau }(u)=\{\begin{array}{cc} \tau u, & \mathrm{if}\;u\geq 0,\\ (\tau -1)u, & \mathrm{if}\;u<0. \end{array}$$

For τ=0.5, this reduces to median regression, which minimizes the sum of absolute deviations rather than squared deviations, providing robustness against outliers and skewed data [3,11,12].

### 1.4. Interpretation of Quantile Regression Coefficients

In ordinary regression, the estimated coefficients describe how the *mean* of Y  changes with a one-unit increase in a predictor $X_{j}$, holding other variables constant. In quantile regression, however, the coefficient $\beta_{j}\left(\tau \right)\;$ expresses how the τ*-th* conditional quantile of Y changes with the same unit increase in $X_{j}$. When the estimated coefficients $\beta_{j}\left(\tau \right)\;$ are similar across quantiles, the predictor exerts a uniform effect across the outcome distribution, and ordinary regression provides an adequate summary. However, when these coefficients differ across quantiles, the predictor’s influence is heterogeneous, revealing differential effects across low, median, and high outcome values [8,13,14]. This property makes quantile regression particularly valuable for epidemiological and clinical data, where skewness, outliers, and heteroscedasticity are the rule rather than the exception [1,5,15].

Moreover, distancing the interpretation of quantile regression from linear models and integrating it into the logic of ANOVA, median regression can be viewed as a multivariable extension of the median test, functioning as a non-parametric analysis of variance on medians rather than means. Unlike the classical median test, which compares medians across groups without adjusting for confounders, median regression enables such comparisons while controlling for multiple covariates. This feature makes it conceptually similar to an ANCOVA model, but one that targets the median of the outcome distribution. Hence, it provides a powerful and flexible framework for assessing differences between groups in the presence of skewed data or unequal variances [16,17,18].

Furthermore, quantile regression represents one of several robust approaches for analyzing non-normally distributed outcomes in epidemiology. Alternative methods include M-estimation and other robust regression techniques, transformation-based models (e.g., Box-Cox regression), and flexible semi-parametric frameworks such as generalized additive models. These approaches address violations of linear regression assumptions in different ways; quantile regression is highlighted here for its ability to model heterogeneity across the outcome distribution.

Beyond the epidemiological focus of this article, it is important to note that ordinary linear regression is widely used across many scientific domains—including economics, psychology, education research, environmental sciences, and biomedicine—where continuous outcomes are routinely modeled under assumptions of normality and homoscedasticity. While the present study employs an epidemiological dataset for illustrative purposes, the methodological issues discussed here extend broadly across these fields as well. Both ordinary linear regression and quantile regression are applicable in diverse research settings, and the epidemiological context is used primarily for its familiarity and pedagogical clarity in demonstrating the advantages of distribution-sensitive modeling.

## 2. Application Example in Epidemiology: The Body Mass Index (BMI)

Body mass index (BMI) is commonly used as the main indicator of obesity and is a key determinant of metabolic and cardiovascular risk [19,20]. Interindividual variation in BMI reflects the combined effects of demographic, behavioral, and lifestyle characteristics, among which sex, age, energy intake, physical activity, and dietary patterns are consistently identified as primary influences on body weight regulation [21].

To examine the complex relationships between BMI and these five determinants, and to uncover aspects that may not be apparent in mean-based models, both ordinary least squares regression and quantile regression were applied.

### 2.1. Ordinary Least Squares Regression and Quantile Regression

Ordinary regression estimates the mean effect of each predictor on BMI, assuming that the relationships remain constant across the entire BMI distribution and that residuals are normally distributed with equal variance [22]. Quantile regression, in contrast, enables the estimation of covariate effects at different points of the BMI distribution, thereby revealing potential heterogeneities that are not detectable by conventional approaches. While useful for summarizing average trends, this approach may obscure important variations in predictor effects among individuals at different levels of BMI [7,8,10]. To address this limitation, quantile regression is applied as a complementary approach. By estimating conditional quantiles of BMI, it allows the evaluation of whether the effects of sex, age, energy intake, and physical activity differ across the distribution of BMI [1,15,23]. In this study, quantile regression was estimated at the 25th, 50th, 75th, and 90th percentiles. These quantiles were chosen because they span the lower, central, and upper regions of the BMI distribution, a convention widely used in methodological demonstrations. In addition, the empirical values of these quantiles correspond approximately to WHO BMI ranges in this dataset, thereby providing clinically interpretable contrasts. Although alternative quantiles aligned exactly with WHO cutoffs could be selected, the current choices serve the pedagogical aim of illustrating distributional heterogeneity across multiple regions of the BMI distribution. Comparing the ordinary and quantile regression results provides a more comprehensive understanding of how these factors influence body weight—revealing patterns that might otherwise remain hidden when analysis is confined to mean-based models [5,6,14].

### 2.2. Data Selection and Collection

The present work is a secondary analysis based on data collected from an epidemiological study conducted among 1415 apparently healthy Greek men and women aged 25–82 years. All participants had provided written informed consent prior to enrollment. Anthropometric, dietary, and lifestyle data were reanalyzed to examine associations between BMI and its main determinants. The original population-based survey was conducted between 1991–1993 using multistage sampling across several Greek regions. Although designed to capture dietary patterns at the national level, the sample may not fully represent the contemporary Greek population. These data were used solely for secondary methodological demonstration.

Dietary information was collected using a validated semi-quantitative food frequency questionnaire designed to assess the average frequency of consumption of a broad range of foods and beverages over the preceding year. Standard portion sizes were used to estimate food quantities, and nutrient and energy intakes were calculated using a food composition database specifically adapted to the Greek diet [24,25]. Intakes were expressed as daily averages, accounting for seasonal variation in food consumption [26].

Participants also provided data on physical activity (yes/no) and current dieting status (yes/no) for health or aesthetic reasons. Physical activity and dieting status were originally collected as binary variables (‘yes/no’) in the questionnaire. Accordingly, their inclusion in the models as binary covariates does not involve any dichotomization of continuous information and reflects the structure and intent of the original instrument. Anthropometric measurements, including weight, height, and waist and hip circumferences, were taken using standardized procedures, with participants measured without shoes and in light clothing.

### 2.3. Data Analysis

Descriptive statistics were calculated for all study variables. Continuous variables are presented as means and standard deviations (SD), while categorical variables are presented as frequencies and percentages. The associations between body mass index (BMI) and its main determinants—sex, age, energy intake, physical activity, and dietary status—were examined using both ordinary least squares regression and quantile regression models.

The ordinary model was applied to estimate the mean effect of each predictor on BMI, under the assumptions of normality and homoscedasticity of residuals. The assumption of normality was assessed using graphical diagnostic tools, including the normal probability (Q–Q) plot and the histogram of standardized residuals. To explore potential heterogeneity across the BMI distribution, quantile regression was employed at selected percentiles (the 25th, 50th, 75th and 90th), allowing the estimation of covariate effects at different points of the conditional BMI distribution.

Sex and physical activity were entered in the models as binary variables (male vs. female; yes vs. no), while age (in years) and energy intake (measured per 100 kcal) were treated as continuous predictors. Dietary status (yes vs. no), indicating whether participants were following any type of diet for health or aesthetic reasons, was also included as a binary covariate.

The comparison between ordinary and quantile regression results enabled the evaluation of whether the relationships between BMI and its determinants were homogeneous across all levels of BMI or varied between leaner and heavier individuals. Particular emphasis was placed on comparing the two methods in terms of the type and depth of information each provides, highlighting how quantile regression may reveal distributional effects that remain undetected in mean-based models. Statistical analyses were conducted using IBM SPSS Statistics, version 28 (IBM Corp., Armonk, NY, USA) [27]. Quantile regression in SPSS was estimated using an enhanced interior-point optimization algorithm, which is designed for stable computation in large datasets. Standard errors for quantile regression estimates were computed using the asymptotic Huber–Sandwich estimator. Confidence intervals were set at the 95% level. Although this implementation is efficient and appropriate for the purposes of the present analysis, other statistical environments, such as R 4.5.2 (e.g., the ‘quantreg’ package) and Python 3.14.2 (e.g., ‘statsmodels’ module), offer more flexible quantile regression frameworks that allow users to choose among alternative optimization algorithms (e.g., simplex, interior-point, smoothing methods) and provide extended functionality for inference, diagnostics, and model customization.

## 3. Results

Table 1 summarizes the frequencies, mean values, and standard deviations of the predictor variables included in the regression analyses, stratified by intervals defined by the three quartiles (Q_0.25_, Q_0.50_, Q_0.75_) and the Q_0.90_ quantile of BMI. These quantiles broadly delineate the distribution of BMI across normal and abnormal ranges: Q_0.25_ = 24.7 kg/m^2^, Q_0.75_ = 30.4 kg/m^2^, and Q_0.90_ = 33.6 kg/m^2^. According to the World Health Organization (WHO) classification (WHO, 2000), BMI values < 25 kg/m^2^ indicate normal weight, 25–29.9 kg/m^2^ overweight, 30–34.9 kg/m^2^ class I obesity, 35–39.9 kg/m^2^ class II obesity, and ≥40 kg/m^2^ class III obesity. In the present analysis, BMI values below the first quartile (<Q_0.25_; <24.7 kg/m^2^) correspond to the normal range; values within the interquartile range (Q_0.25_–Q_0.75_; 24.7–30.4 kg/m^2^) indicate overweight status; values between the third quartile and Q_0.90_ (Q_0.75_–Q_0.90_; 30.5–33.6 kg/m^2^) represent, broadly, class I obesity; and values above Q_0.90_ (>33.6 kg/m^2^) correspond to class II or III obesity. Regression models will be estimated for these specific quantiles and additionally the median (Q_0.25_, Q_0.50_, Q_0.75_, and Q_0.90_), to capture variations across the BMI distribution.

From Table 1, a positive linear association between BMI and age, and a negative association between BMI and physical activity, are evident. The relationships with the remaining variables appear more complex, with no consistent pattern emerging. Notably, marked gender differences are observed at higher BMI levels: while overweight and class I obesity are more prevalent among men, the proportions reverse at higher degrees of obesity. Specifically, 18.5% of men and 29.6% of women fall within the normal weight range, whereas in high-class obesity (>Q_0.90_; BMI > 33.6 kg/m^2^) the proportion of women (12.8%) is approximately double that of men (6.1%).

**Figure 3:**
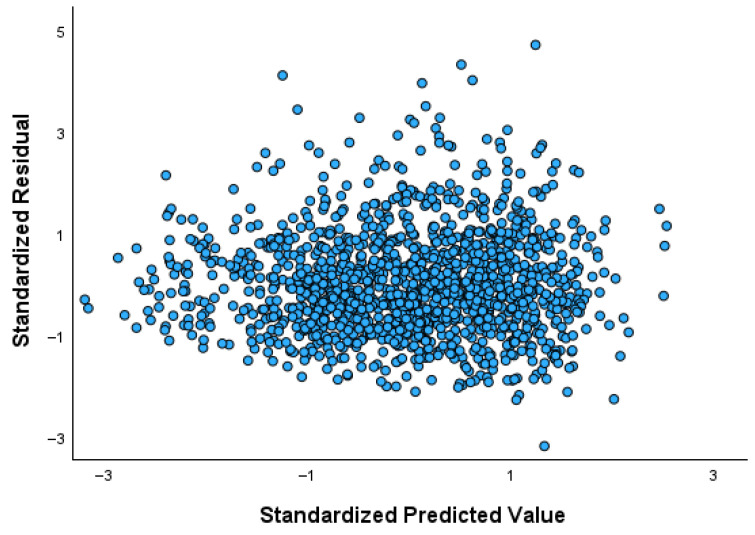
The residuals-versus-fitted plot of the ordinary regression model on BMI.

Table 2 presents the results of the ordinary linear regression model with BMI as the dependent variable and gender, age, physical activity, dieting, and daily energy intake (measured per 100 kcal) as predictors. Age and daily energy intake are positively associated with BMI, whereas physical activity shows a negative association. No clear or monotonic relationship with BMI is observed for the remaining independent variables. Figure 3 illustrates the residuals-versus-fitted plot, revealing a violation of the homoscedasticity assumption, as indicated by the conical dispersion pattern of the residuals. This widening pattern reflects substantial heteroscedasticity, thereby supporting the use of quantile regression, which is not affected by non-constant variance.

Four quantile regression models were then run for the BMI quantiles Q_0.25_, Q_0.50_, Q_0.75_, and Q_0.90_. The results are summarized in Table 3.

Across the three quartiles (Q_0.25_, Q_0.50_, Q_0.75_), the negative association between BMI and physical activity, and the positive associations with age and energy intake, are consistently observed. At Q_0.90_, energy intake remains positively associated with BMI but loses statistical significance. The non-significance at Q_0.90_ likely reflects reduced precision and larger standard errors typically observed at the upper tail of the distribution, rather than the absence of a biological association. The coefficients for physical activity across the four quantile models are −1.05, −1.46, −1.77, and −2.42. This gradient in coefficients indicates that BMI is systematically lower among physically active individuals compared to those who are inactive throughout the entire distribution. Furthermore, the difference in BMI between active and inactive individuals tends to widen at higher quantiles, with a difference of 2.42 kg/m^2^ at Q_0.90_ (higher among adults who do not exercise). The differences are relatively smaller in the lower and central quantiles of BMI.

Gender exhibits a particularly interesting pattern not shown by ordinary regression, as the differences between men and women are not uniform across the BMI distribution. The first quartile and the median (Q_0.25_ and Q_0.50_) for women are 0.73 and 0.43 kg/m^2^ lower, respectively, than those of men, whereas Q_0.75_ and Q_0.90_ are 0.60 and 2.14 kg/m^2^ higher, respectively. This suggests that overweight is more prevalent among men, while higher degrees of obesity are more common among women.

**Figure 4:**
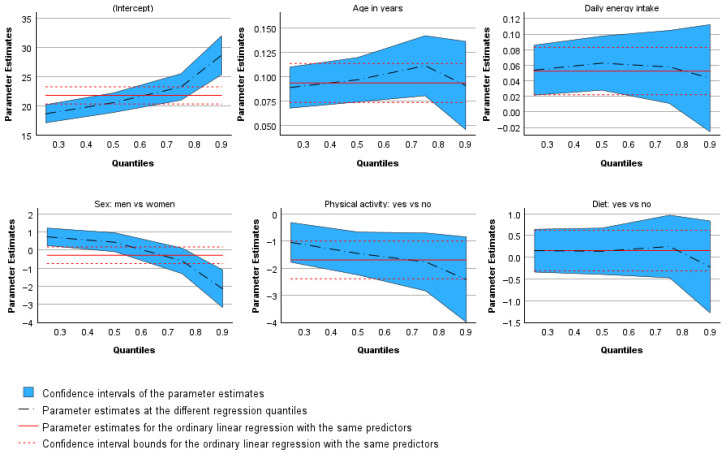
Plots of the estimated parameters. Shaded areas represent 95% confidence intervals.

The plots of the estimated parameters [28] that follow illustrate the variation in coefficients across all quantile regressions (Figure 4). In the plots for age, energy intake, physical activity, and dieting, the dashed line representing the estimated coefficients remains within the confidence intervals of the corresponding parameters from the ordinary least squares regression. This indicates that the results for these variables do not differ substantially from those of the linear regression model. Only the line representing the gender coefficients lies outside the confidence interval range, graphically confirming the earlier finding that the effect of gender on BMI is not monotonic in direction. The regression coefficients for the median and the 0.75 quantile fall within the confidence interval boundaries, whereas those for the 0.25 and 0.90 quantiles alternate in sign, lying below and above the confidence region, respectively.

## 4. Discussion

This study highlights the methodological advantages of quantile regression compared with the conventional ordinary linear approach using epidemiological data (i.e., BMI) as an example of outcomes, which frequently exhibit skewed distributions, outliers, and heteroscedasticity [7,8,10,29]. Ordinary linear regression estimates the conditional mean of an outcome under the assumptions of normally distributed and homoscedastic residuals. When these assumptions are violated—a frequent occurrence in biomedical and public health research—the estimated parameters may fail to capture important heterogeneity across the outcome distribution [4,30,31]. In contrast, quantile regression relaxes these restrictive assumptions by modeling conditional quantiles rather than conditional means, thereby allowing the estimation of predictor effects across the entire range of the outcome variable [12,32,33]. This framework provides a distribution-sensitive analytical perspective that reveals non-uniform associations and subpopulation-specific effects that mean-based models are unable to detect [1,15,34]. In the present analysis, for example, while ordinary regression identified average positive associations of BMI with age and energy intake and a negative association with physical activity, quantile regression exposed the non-linearity and variation in these relationships across the BMI distribution. The inverse association with physical activity intensified progressively toward higher BMI quantiles, and the gender effect reversed direction at the upper tail. Such findings illustrate the added value of quantile regression in identifying complex, non-homogeneous relationships that may be masked when only mean effects are examined [13,35].

Beyond its robustness to non-normality and outliers, quantile regression offers several additional methodological advantages. It enables the examination of how determinants influence not only the central tendency but also the lower and upper tails of an outcome distribution—regions that often correspond to clinically or socially significant phenomena [1,35]. This characteristic is particularly valuable in epidemiological contexts, where the mechanisms underlying extreme outcomes (e.g., severe obesity, prolonged hospitalization, or elevated healthcare costs) may differ substantially from those shaping average outcomes [2,30,36]. Furthermore, quantile regression facilitates direct comparisons with ordinary linear estimates through graphical tools such as quantile coefficient plots, providing an intuitive visualization of distributional heterogeneity [3,31].

Taken together, the above observations emphasize that the choice between regression approaches should be guided by both the research objective and the underlying data structure. Ordinary linear regression remains appropriate when the primary goal is to estimate average associations under approximately normal and homoscedastic conditions. In contrast, when the focus shifts toward understanding variability, identifying subgroup-specific effects, or analyzing outcomes characterized by skewness or outliers, quantile regression offers a more informative and flexible alternative. By modeling the entire conditional distribution rather than only its mean, quantile regression complements the ordinary regression framework, yielding a richer, distribution-sensitive understanding of population heterogeneity [37].

An additional strength of quantile regression lies in its median formulation (the 0.50 quantile), which can be interpreted as a multivariate median regression [8,16]. This formulation extends the traditional median test into a regression-based, multivariable framework—effectively functioning as a nonparametric analogue of ANCOVA on medians rather than means. In doing so, it bridges parametric and nonparametric inference, combining the robustness and interpretive clarity of median-based statistics with the flexibility of regression modeling [17,33]. This perspective reinforces the role of quantile regression as a methodological bridge between classical parametric analysis and distributional modeling, offering a conceptually coherent framework for exploring heterogeneity under minimal statistical assumptions.

Beyond the methodological distinctions between ordinary and quantile regression, the present article also serves an educational purpose. It was designed to illustrate, through a simple epidemiological example, the conceptual foundations and interpretive potential of quantile regression. Rather than emphasizing the empirical findings per se, the analysis aims to demonstrate how quantile regression can be applied and why it may yield richer insights than traditional linear models. In this respect, the article functions as a methodological guide for applied researchers—particularly for those without formal training in advanced statistical modeling—by providing a transparent, intuitive, and reproducible example of the use of quantile regression in population health research [1,15,29]. This pedagogical focus underscores the value of quantile regression not only as a robust analytical method but also as a bridge between statistical theory and practical application in epidemiology and public health.

More broadly, quantile regression has been widely adopted in several scientific domains where distributional heterogeneity is central to inference. In economics, it is routinely used to study wage structures and income inequality; in environmental sciences, to model climatic extremes and pollutant concentration distributions; and in ecology and social sciences, to investigate skewed patterns such as species abundance and socioeconomic disparities. These applications collectively highlight the versatility of quantile regression as a distribution-sensitive analytical tool capable of addressing research questions that cannot be adequately explored using mean-based models alone [4,38,39,40].

Future methodological work should further explore quantile regression extensions, such as mixed-effects and Bayesian formulations, which could enhance its applicability in longitudinal and clustered epidemiological data [12,14,41,42,43,44].

## 5. Conclusions

Quantile regression offers a conceptually coherent and empirically flexible framework for epidemiological data analysis. By estimating conditional quantiles rather than conditional means, it enhances the understanding of population variability and uncovers heterogeneity often overlooked by ordinary regression. The method relaxes traditional assumptions, accommodates non-normality and heteroscedasticity, and extends median-based reasoning into a multivariate analytical context.

As demonstrated in this study, quantile regression complements rather than replaces traditional approaches, providing distribution-sensitive insights that improve the interpretive precision of epidemiological investigations. Broader adoption of quantile regression could strengthen the analytical foundations of population health research by identifying heterogeneous effects and subgroup-specific risk mechanisms that mean-based models may fail to capture.

Although illustrated here with an epidemiological example, the methodological strengths of quantile regression are equally relevant across other fields—such as economics, environmental studies, ecology, and the social sciences—where understanding effects across the entire outcome distribution is often essential. Presented in an applied and accessible context, this paper aims to encourage the wider use of quantile regression among applied researchers across public health and other scientific fields, positioning it both as a robust analytical tool and as an educational bridge toward more distribution-aware approaches to data analysis.

## Figures and Tables

**Table 1:** Distribution of study variables across BMI quantiles.

			<Q_0.25_		Q_0.25_–Q_0.50_		Q_0.50_–Q_0.75_		Q_0.75_–Q_0.90_		>Q_0.90_	
BMI (kg/m^2^)			<24.7		24.7–27.4		27.5–30.4		30.5–33.6		>33.6	
	N	%	Ν	%	Ν	%	Ν	%	Ν	%	Ν	%
			353	24.9	354	25.0	355	25.1	212	15.0	141	10.0
Sex ^1^												
Men	593	41.9	110	18.5	171	28.8	186	31.4	90	15.2	36	6.1
Women	821	58.1	243	29.6	183	22.3	169	20.6	122	14.8	105	12.8
Physical Activity ^2^												
Yes	174	12.3	70	40.2	56	32.2	32	18.4	13	7.5	3	1.7
No	1241	87.7	283	22.8	298	24.0	323	26.0	199	16.0	138	11.1
Diet ^3^												
Yes	498	35.2	113	22.7	122	24.5	130	26.1	85	17.1	48	9.6
No	917	64.8	240	26.2	232	25.3	225	24.5	127	13.8	93	10.1
												
	mean	SD	mean	SD	mean	SD	mean	SD	mean	SD	mean	SD
Age in years ^4^	52.9	11.9	48.7	11.9	51.3	10.8	54.6	11.8	56.5	11.5	57.4	11.4
Energy (in kcal) ^5^	2458	780	2438	749	2458	754	2515	780	2429	776	2410	918

^1^ *p* < 0.001, ^2^ *p* < 0.001, ^3^ *p* = 0.369: *χ*^2^ test; ^4^ *p* < 0.001, ^5^ *p* = 0.567: One-way ANOVA for linear trend.

**Table 2:** Ordinary regression analysis results. (*R*^2^ = 0.09, *adjR*^2^ = 0.08).

	*β*	*p* Value	95% CI
Sex (men vs. women)	−0.29	0.218	(−0.75, 0.17)
Physical activity (yes vs. no)	−1.70	<0.001	(−2.39, −1.00)
Diet (yes vs. no)	0.16	0.516	(−0.31, 0.62)
o	0.09	<0.001	(0.07, 0.11)
Energy (per 100 kcal)	0.05	<0.001	(0.02, 0.08)

**Table 3:** Quantile regression model results.

	Q_0.25_ (Pseudo *R*^2^ = 0.06)			Q_0.50_ (Pseudo *R*^2^ = 0.06)		
	*β*_25_	*p* value	95% CI	*β*_50_	*p* value	95% CI
Sex (men vs. women)	0.73	0.003	(0.25, 1.22)	0.43	0.106	(−0.09, 0.96)
Physical activity (yes vs. no)	−1.05	0.005	(−1.78, −0.32)	−1.46	<0.001	(−2.25, −0.67)
Diet (yes vs. no)	0.15	0.545	(−0.34, 0.65)	0.14	0.610	(−0.40, 0.67)
Age in years	0.09	<0.001	(0.07, 0.11)	0.10	<0.001	(0.07, 0.12)
Energy (per 100 kcal)	0.05	0.001	(0.02, 0.09)	0.06	<0.001	(0.03, 0.10)
	Q_0.75_ (Pseudo *R*^2^ = 0.05)			Q_0.90_ (Pseudo *R*^2^ = 0.06)		
	*β*_75_	*p* value	95% CI	*β*_90_	*p* value	95% CI
Sex (men vs. women)	−0.60	0.096	(−1.31, 0.11)	−2.14	<0.001	(−3.18, −1.10)
Physical activity (yes vs. no)	−1.77	0.001	(−2.84, −0.70)	−2.42	0.003	(−3.99, −0.85)
Diet (yes vs. no)	0.25	0.499	(−0.47, 0.97)	−0.22	0.680	(−1.28, 0.84)
Age in years	0.11	<0.001	(0.08, 0.14)	0.09	<0.001	(0.05, 0.14)
Energy (per 100 kcal)	0.06	0.016	(0.01, 0.11)	0.04	0.217	(−0.3, 0.11)

## Data Availability

The data presented in this study are available on request from the corresponding author.
